# Corrected Version: Sodium Benzoate, A Metabolite of Cinnamon and A Food Additive, Improves Cognitive Functions in Mice After Controlled Cortical Impact Injury

**DOI:** 10.33140/jcei.11.01.03

**Published:** 2026-03-30

**Authors:** Suresh B. Rangasamy, Sumita Raha, Sridevi Dasarathi, Kalipada Pahan

**Affiliations:** 1 Division of Research and Development, Jesse Brown Veterans Affairs Medical Center, Chicago, USA; 2 Department of Neurological Sciences, Rush University Medical Center, Chicago, USA

**Keywords:** Sodium Benzoate, TBI, Glial Activation, Lesion Cavity, Memory and Learning, Neuroprotection, Controlled cortical impact (CCI), Neuroinflammation, Microglia, iNOS, Lesion volume, Cognitive and Motor Function, Drug Repurposing

## Abstract

Traumatic brain injury (TBI) is a major health concern, sometimes leading to long-term neurological disability, especially in children, young adults, and war veterans. Although the research investigators and clinicians have applied different treatment strategies or neurosurgical procedures to solve this health issue, we are still in need of effective therapy to halt the pathogenesis of brain injury. Earlier we have reported that sodium benzoate (NaB), a metabolite of cinnamon and a Food and Drug Administration-approved drug against urea cycle disorders and glycine encephalopathy, protects neurons in animal models of Parkinson’s disease and Alzheimer’s disease. This study was undertaken to examine the therapeutic efficacy of NaB in controlled cortical impact (CCI)-induced preclinical mouse model of TBI. Oral treatment with NaB, but not sodium formate (NaFO), was found to decrease the activation of microglia and astrocytes and inhibits the expression of inducible nitric oxide synthase (iNOS) in hippocampus and cortex of CCI-insulted mice. Further, administration of NaB also reduced the vascular damage and decreased the size of lesion cavity in the brain of CCI-induced mice. Importantly, NaB-treated mice showed significant improvements in memory and locomotor functions as well as displayed substantial reduction in depression like behaviors. These results delineate a novel neuroprotective property of NaB, highlighting its possible therapeutic importance in TBI.

## 1. Introduction

In the 2021 article by Rangasamy et al., images of the bottom panel (CA1) of Figure 2C and both the panels (cortex and CA1) of Figure 2D had overlaps with other images [1]. Therefore, the article [1] was retracted. Now, we have corrected these honest mistakes in the current manuscript.

Traumatic brain injury (TBI) is a leading cause of disability and death, particularly in children and young adults in the United States [2,3]. Nearly 1.5 million people suffer from a TBI every year, approximately 50,000 of them die from TBI related complications each year and 85,000 suffer from long term disabilities [4]. Based on the CDC report (United States Centers for Disease Control and Prevention), the leading cause of TBI are falls (28%), hitting with an object (19%), motor vehicle crashes (20%), assaults (11%) and others (12%). In addition to serious personal issues in health, the costs to society for care and lost productivity due to TBI are huge and estimated at $76.5 billion annually [5,6]. Most of the TBI survivors suffer from different clinical symptoms, such as depression, cognitive/memory deficits, epilepsy and motor function impairments throughout the rest of their lives.

Following TBI, a series of complex pathophysiological events occurs, causing both structural damage and functional deficits [2,3]. Activation of glial cells and associated upregulation of proinflammatory molecules in the CNS participate in the pathogenesis of a number of neurodegenerative and neuroinflammatory diseases [7–10]. Accordingly, one of the main hallmarks of both acute and chronic TBI is also neuroinflammation, which is evidenced within minutes of TBI [11–14]. Studies from laboratory animals of focal and diffuse TBI have shown the involvement of various proinflammatory molecules such as IL-1β, TNF-α and inducible nitric oxide synthase (iNOS) in the pathogenesis of TBI [10,11,13–15]. Many clinical studies demonstrated the increases in IL-1β and TNF-α in CSF and serum of TBI patients as compared to healthy controls [16,17]. Upregulation of broad-spectrum proinflammatory molecules in the brain causes edema, BBB leakage, neuronal apoptosis and atrophy, eventually leading to functional impairments [18].

Sodium benzoate (NaB), the sodium salt of the simplest aromatic carboxylic acid, is a widely used food preservative and a metabolite of cinnamon [19–22]. NaB is also an FDA approved drug against severe metabolic defects such as urea cycle disorders and glycine encephalopathy or non-ketotic hyperglycinemia [23,24]. It has been reported that 2% solution of NaB in drinking water is safe for lifelong treatment in mice without any noticeable side effects [25]. Our lab has delineated that NaB treatment through drinking water is capable of protecting mice from relapsing remitting EAE [26,27] probably via STAT6-mediated upregulation of transforming growth factor β (TGFβ) and regulatory T cells [28]. NaB treatment also upregulates neuroprotective proteins and protects dopaminergic neurons in a mouse model of Parkinson’s disease (PD) [29,30]. In a mice study, we have also evidenced that oral treatment of cinnamon and NaB converts poor learners to good learners via upregulation of CREB and increased spatial memory consolidation [31]. Here, we investigated the neuroprotective effect of NaB in a mouse model of TBI and demonstrate that NaB is capable of reducing glial inflammation, decreasing lesion volume and improving cognitive, social and locomotor behaviors in controlled cortical impact (CCI)-induced TBI mice.

## 2. Materials and Methods

### 2.1. Animals

Male C57BL6 mice (7-8 weeks old) purchased from Harlan, Indianapolis, were used for this study. Animal maintenance and the surgical procedure were conducted in compliance with NIH guidelines of the Care and Use Committee and were approved by the Jesse Brown VA Medical Center Animal Care and Use Committee (protocol # 1498771). Animals were housed in an environment with a stable temperature and a 12 h light-dark cycle. Water and food were provided ad libitum.

### 2.2. Controlled Cortical Impact procedure

**Figure 1:**
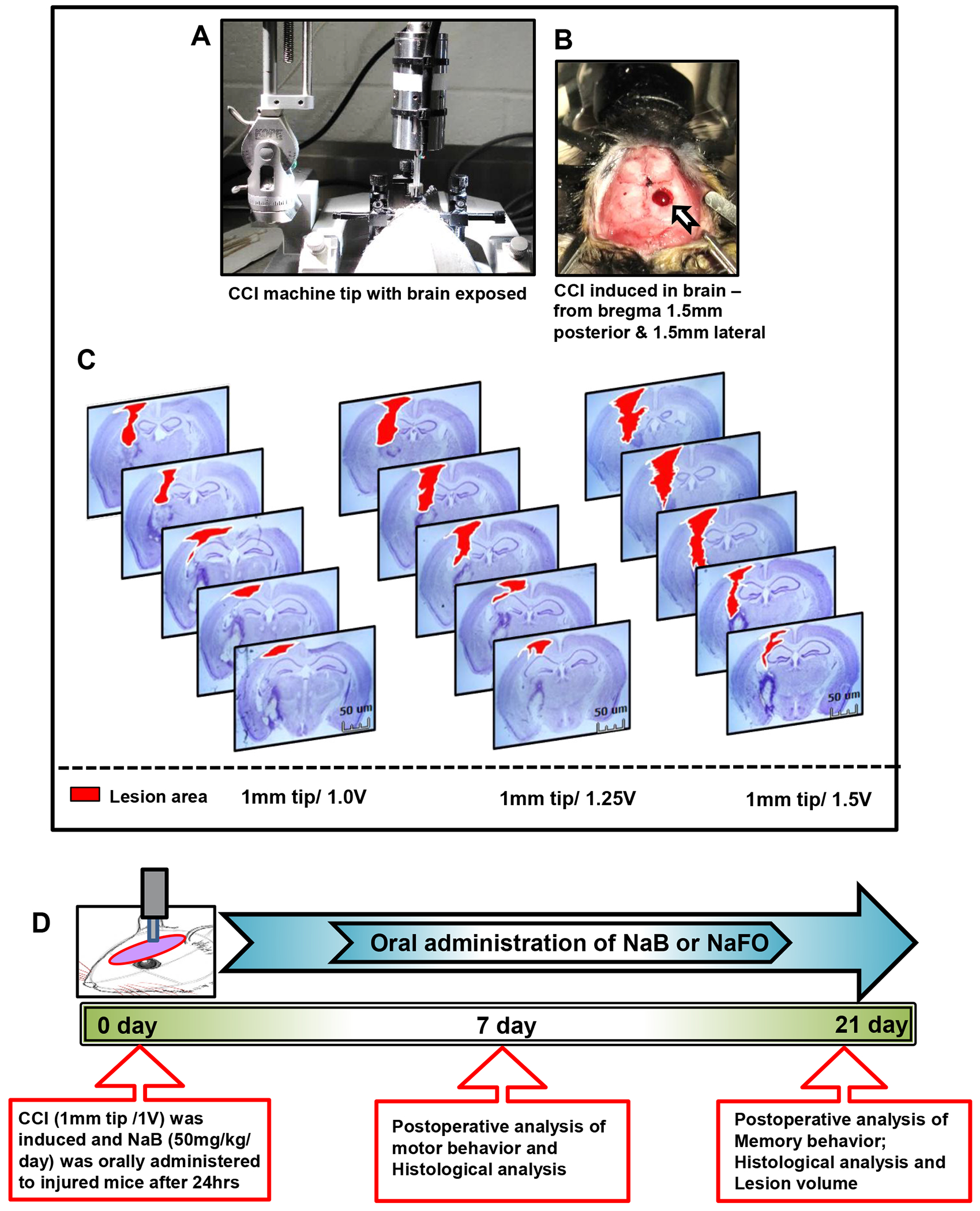
Standardization of CCI-injury parameters and schematic presentation of experiments. (A) Using the CCI technique, brain injury was gently induced onto the exposed brain region of anesthetized mice. In (B), blood clots and tissue damage in a burr hole (stereotactic coordinates—from bregma 1.5 mm posterior and 1.5 mm lateral) were seen in the injured brain region of mice after CCI injury. (C) For the induction of mild, moderate and severe CCI injury, we used a 1 mm tip with three different velocities (V), viz., 1.0 V, 1.25 V and 1.5 V, respectively. After one week post-injury, mice (n = 3) were perfused with 4% paraformaldehyde followed by removal of brains and staining of the brain sections with cresyl violet. (D) Experimental design showing the time course of treatment, and behavioral and histological analysis following CCI injury (1 mm tip/1.0 V). Here, the unit of V is m/s.

To induce brain injury in mice, we applied the controlled cortical impact (CCI) injury technique as described previously [32–34]. Adult C57BL6 mice were anesthetized with 2% isoflurane and allowed to breathe normally without tracheal intubation. Body temperature was maintained at 37°C on a heating pad and monitored by a rectal probe during the surgery. The depth of anesthesia was observed by a gentle toe pinch without causing any injury. The heads of anesthetized mice were shaved with a sterile electric shaver, and skin was cleaned with betadine solutions. Then, the animals’ heads were fixed in a stereotaxic frame and TBI was induced by using the CCI technique (Figure 1A–D). Initially, a midline skin incision was performed to expose the skull, and a 4 mm- diameter craniotomy was performed on the right side of the exposed skull with the coordinates−1.5 mm AP and −1.5 mm ML using the stereotaxic apparatus. Then, the brain was exposed in this burr hole with an intact dura. Under surgical microscope control, a Leica Impact One Stereotaxic Impactor (Leica Microsystems, Buffalo Grove, IL, USA) equipped with a 1.0 mm rounded metal tip was angled vertically towards the brain surface with an intact dura. Subsequently, a mild injury was unilaterally induced with a strike velocity of 1.0 m/s on the right side of the exposed brain region. A sterile sponge immobilization board was used to support the area below the head during the induction of brain injury. After impact injury, the damage was produced in the cerebral cortex, causing extensive structural damage in the surrounding region. Sham group animals underwent a similar surgical procedure but without CCI injury. Then, the operated animal was removed from the stereotaxic holder, and the skin incision was lightly sutured to close the incised region. All operated animals were placed in a thermal blanket for the maintenance of body temperature within the normal limits. These animals were monitored until the recovery from anesthesia and over the next three consecutive postoperative days.

Using small laboratory animals, such as mice, for producing a clinically related TBI model is a challenging task in TBI research. The effect of TBI may vary in physical and psychological outcomes depending on the extent of damage to the brain. Some symptoms may appear immediately after the injury, while others may appear days or weeks later. Therefore, we determined it necessary to use a fixed 1 mm rounded tip with different velocities for standardization purposes. In this study, we randomly divided mice into three groups and applied a 1 mm rounded tip with three velocities, viz., 1.0 V, 1.25 V and 1.50 V, for the induction of mild, moderate and severe injuries, respectively (Figure 1C). At the end of the one-week postoperative period, all three groups of animals were perfused with 4% paraformaldehyde to remove the brain, and subsequently, the brain sections were created with a 40 μm thickness. Using cresyl violet staining, we studied the histopathological features of brain damage that revealed prominent tissue damage in the cortex and hippocampus region in the mild injury group. However, no noticeable damage was seen in the contralateral side of the brain in this group of mice. In the moderate injury group, we found more damage in tissues in the ipsilateral cortex and hippocampus region of mouse brains, and recovery of mice after surgery was found to be extremely slow and fatal in some cases. Further, we noticed serious tissue damage in both the cortex and hippocampus of the ipsilateral side of the brain after surgery in the severe injury group (Figure 1C). Recovery of mice was minimal, and the injury produced became fatal in many cases in this group of mice. Therefore, based on the histopathological observations of three types of injury groups, we decided to use the mild type of CCI injury (1 mm tip and 1.0 V) to delineate the beneficial effects of the cinnamon metabolite NaB in the improvement in cognitive and motor functions after brain injury (Figure 1C).

### 2.3. Treatment with sodium benzoate or sodium formate

NaB and NaFO were solubilized in 0.1% methyl cellulose solution. Starting from 24 hours of CCI injury, mice were orally treated with NaB or NaFO (50 mg/kg/day) once daily for 7 postoperative days. Later, the oral treatment was continued every alternate day till 21 postoperative days and following behavior analysis the mice were sacrificed for histological and biochemical studies.

### 2.4. Experimental groups and NaB/NaFO treatment

Figure 1D shows the experimental design used in this study. All mice were randomized into the following groups:

Group 1: Control/Sham group (n=6 per group): Mice underwent surgery without any injury and treatment.

Group 2: CCI group (n=6 per group): Mice underwent CCI injury and no treatment was carried out.

Group 3: CCI+NaB treatment (n=6 per group): Mice were subjected to CCI and NaB (50 mg/kg/day) treatment orally was started 24 hours after the induction of injury.

Group 4: CCI+NaFO treatment (n=6 per group). Mice were subjected to brain injury and NaFO (50 mg/kg/day) treatment orally was started 24 hours after the induction of injury.

### 2.5. Western Blotting

Western blotting was performed as described in our earlier studies [35–38]. Equal amount of proteins was electrophoresed in 10% or 12% SDS-PAGE and transferred onto nitrocellulose membrane. The blot was probed with primary antibodies overnight at 4°C. The following are the primary antibodies used in this study: anti-iNOS (1:1000, BD Biosciences), anti-Iba1 (1:1000, Abcam), anti-GFAP (1:1000, Santa Cruz Biotechnology, Dallas, TX), and anti-β-actin (1:5000, Abcam) (Table 1). Following the overnight incubation, primary antibodies were removed and the blots were washed with phosphate buffer saline containing 0.1% Tween-20 (PBST) and corresponding infrared fluorophore tagged secondary antibodies (1:10,000, Jackson Immuno- Research) were added at room temperature. The blots were then incubated with secondary antibodies for 1 hour. Later, blots were scanned with an Odyssey infrared scanner (Li-COR, Lincoln, NE). Band intensities were quantified using the ImageJ software (NIH, USA).

### 2.6. Immunohistochemistry

Mice were anesthetized with ketamine-xylazine mix solutions and perfused with PBS and then with 4% paraformaldehyde (w/v) in PBS, followed by dissection of the brain for immunofluorescence microscopic examination [35,36,39]. Briefly, the dissected brains were incubated in 10% sucrose for 3 hours and then followed by 30% sucrose overnight at 4°C. Then the brains were embedded in optimal cutting temperature medium (Tissue Tech) at −80°C and processed for conventional cryosectioning. Frozen sections (40 μm thickness) were treated with cold ethanol (−20°C), washed with PBS, blocked with 2% BSA in PBST, and double labeled with two primary antibodies (Table-1). After three washes with PBST, sections were incubated with Cy2 and Cy5 (Jackson ImmunoResearch Laboratories). The sections were mounted and observed under an Olympus IX81 fluorescence microscope. Counting analysis was performed using Olympus Microsuite V software with the help of a touch counting module.

### 2.7. Quantification of lesion volume using stereological techniques

The estimation of lesion volume was performed based on the Cavalieri method of unbiased stereology using the StereoInvestigator software (MicroBright Biosciences, USA) [35,40]. Both the ipsilateral and contralateral hemisphere of brain volumes were determined using the Cavalieri estimator with a 1 mm grid spacing 1 mm. Every fourth section was analyzed beginning from a random start point. Lesion volume was estimated by subtracting the volume of the ipsilateral hemisphere from that of the contralateral hemisphere. Then the volume of lesion cavity estimated in brain section of untreated mice was compared with lesion volume of brain sections of drug treated mice.

### 2.8. Behavioral analysis

Analysis of behaviors in animals were conducted on the 7th and 21st postoperative days after CCI injury. These time-points for behavioral testing were selected based upon earlier studies with these animal models where behavioral abnormalities were seen at these time points [35,41].

### 2.9. Open field behavior

The performance of animals in open field test was analyzed as described in our earlier studies [10,36,42]. Briefly, each animal was allowed to move freely to explore an open field arena designed with a square shaped wooden floor measuring 40 x 40cm, with walls 30 cm high for 5 min. A video computer 6 (*Basler Gen I Cam - Basler acA* 1300-60) connected to a Noldus computer system was fixed in top facing-down on the open field arena. Each mouse was placed individually on center of the arena and the performance was monitored by the live video tracking system. The central area was arbitrarily defined as a square of 20 x 20cm (half of the total area).

### 2.10. Rotarod

The fore-hindlimb motor coordination and balance in animals was observed using the rotarod test as described in earlier studies [10,43,44]. Briefly, each mouse was placed on the confined section of the rod and trial was initiated with a smooth increase in speed from 4 rpm to 40 rpm for 5 mins. If the mouse did not fall from the rod, it was removed from the rod after 5 mins. The latency to fall was measured in seconds and used for the analysis. Following the CCI injury, each mouse performed the task three trials during the testing sessions and the average score on these three trials was used as the individual rotarod score. Each trial on the rod was terminated when the mice fell off the rod or held on to the rod by hanging and completed improper revolutions.

### 2.11. Tail suspension test

Mice were subjected to the tail suspension test using a methodology as described in earlier studies [10,45,46]. The mice were gently hung upside down by the tail using the non-toxic adhesive tape 50 cm above the floor for 6 mins. Immobility time was defined as the period of time during which the mice only hung passively, without any active movements. An increased immobility time is defined as a depression-like behavior.

### 2.12. Nesting behavior

This test was performed as described in earlier studies [10,47,48]. Briefly, a nestlet consisting of a 5 cm x 5 cm pressed cotton square was kept inside the cage between 5 pm. and 6 pm. Next morning between 9 am. to 10 am, two observers blind to our experimental procedures scored the quality of nest built by the mice using a 5-point scale as follows: Score 1 (> 90% of nestlet intact), Score 2 (50% to 90% of nestlet intact), Score 3 (10% to 50% of nestlet intact but no recognizable nest site), Score 4 (<10% of nestlet intact, nest is recognizable but flat), Score 5 (<10% of the nestlet intact, nest is recognizable with walls higher than the mouse body).

### 2.13. Beam runway

The beam runway made of smooth wooden material and measures 65 cm length x 0.7 cm breadth x 4 cm height. A black box with an opening was fixed at one end and an aversive stimulus (bright lamp) at the other end of beam. This test was used to evaluate the complex coordination and balance of mice while traversing the beam and we performed the procedure as described in earlier studies [10,49]. The mouse was placed on the beam near the light source and the light was turned ‘on’ and this makes the animal move into the box to avoid the aversive stimulus, which was then turned off. Six repetitions were performed with a 2 mins resting period inside the box. The parameters measured were the time taken (sec) to reach the box and the number of steps with contralateral limb drag/slips. An error was considered whenever the paw slipping on the beam and the number of slips were counted. The beam walk analysis was performed by an observer blinded to the treatment at 7th and 21st postoperative day.

### 2.14. Grid runway

The grid runway (65cm length x 8 cm breadth x 1 cm intervals) made of parallel grid bars with interbar intervals of 1 cm apart and grid were kept above the surface on a table during the testing session [10,49]. The soft padding was positioned under the grid runway in the event for protection to avoid serious injury, if the animal falls from the grid. Each mouse was allowed to walk freely on grid and the time taken and number of steps to cross the runway was noted. Each successful foot placement on grid was recorded as a step. However, an error was considered whenever the paw slips through the grid or the paw misses a bar and extends downwards through the plane of bars. The locomotor behavior of animal on grid was evaluated by an observer blinded to the treatment on 7th and 21st day after CCI injury.

### 2.15. Barnes maze test

The Barnes maze test was performed as described in our earlier studies [10,35,50]. Briefly, the mice were initially trained for 2 consecutive days followed by examination on day 3. After each training session, maze and escape tunnel were thoroughly cleaned with a mild detergent to avoid instinctive odor avoidance due to mouse’s odor from the familiar object. On day 3, a video camera (*Basler Gen I Cam - Basler acA* 1300-60) connected to a *Noldus* computer system was placed above the maze and was illuminated with high voltage light that generated enough light and heat to motivate animals to enter into the escape tunnel. The performance was monitored by the video tracking system (*Noldus System*). Cognitive behavior parameters were examined by measuring latency (duration before all four paws were on the floor of the escape box) and errors (incorrect response before all four paws were on the floor of the escape box).

### 2.16. T-maze

The T-maze test was conducted as previously described [10,51]. Mice were initially habituated in the T-maze for 2 days under food- deprived conditions. Food reward was provided for at least 5 times over a 10 mins period of training. T-maze was cleaned with mild detergent solution between each testing session, so as to minimize the animal’s ability to use any olfactory clues. The food- reward side was always associated with a visual cue. Each time the animal consumed food-reward and it was considered as a positive turn.

### 2.17. Novel object recognition (NOR) test

This test evaluates the animal’s ability to recognize the novel object in the environment and monitor short-term memory as described in our earlier studies [10,51]. Initially, the mice were placed in a square novel box (20 in. long x 8 in. high) surrounded with an infrared sensor. Two plastic toys (2.5-3 in. size) that varied in color, shape, and texture were placed in specific locations in the environment 18in. away from each other. The mice were able to freely explore the environment and objects for 15 mins and were then placed back into their individual home cages. After 30 mins intervals, the mice were placed back into the environment, with the 2 objects in the same locations, but now one of the familiar objects was replaced with a third novel object. The mice were again allowed to freely explore both objects for 15 mins. The familiar and novel objects were thoroughly cleaned with a mild detergent after each testing session.

### 2.18. Statistical analysis

Based on our previous studies of similar types and complexities, six mice were expected to return> 80% power for all behavioral experiments. Statistical analyses were performed with Student’s t-test for two-group comparisons, and one-way ANOVA or two-way ANOVA followed by Tukey’s post hoc tests using GraphPad Prism 8. Data are represented as mean ± SD. Statistical significance was determined at the level of p < 0.05 [35,52].

## 3. Results

### 3.1. NaB treatment attenuates glial activation in CCI-induced TBI mice

**Figure 2:**
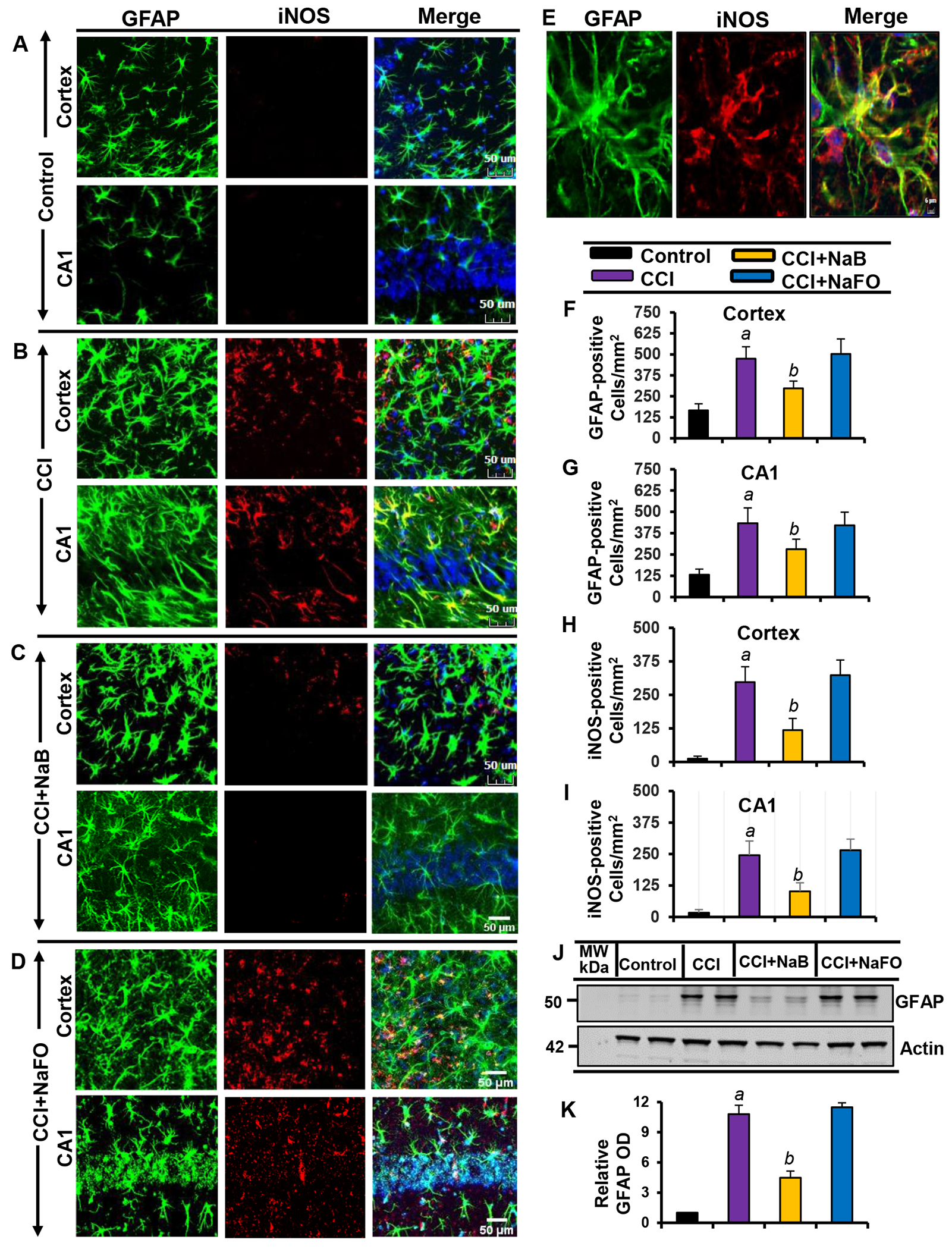
Oral treatment of NaB attenuates the activation of astrocytes *in vivo* in the cortex and hippocampus region of mice with CCI injury. Mice were treated with 50 mg/kg/day of NaB or NaFO via oral administration after the induction of CCI injury. After 7 days of NaB treatment, brain sections were analyzed by double-label immunofluorescence for GFAP and iNOS (A, control; B, CCI injury; C, CCI+NaB; D, CCI+NaFO). (E) Confocal image demonstrating the co-localization of iNOS and GFAP in the cortex. Cells positive for GFAP were counted in the cortex (F) and CA1 region (G). Subsequently, iNOS-positive cells were also counted in the cortex (H) and CA1 region (I). Results represent analysis of six sections of six mice each per group. *^a^ p* < 0.001 vs. control; *^b^ p* < 0.001 vs. CCI injury. Tissue extracts of hippocampal region from all groups of mice (n = 4 per group) were immunoblotted for GFAP (J). Actin was run as a loading control. (K) Bands were scanned, and values (GFAP/Actin) are presented as relative to control. *^a^ p* < 0.001 vs. control; *^b^ p* < 0.001 vs. CCI injury.

**Figure 3:**
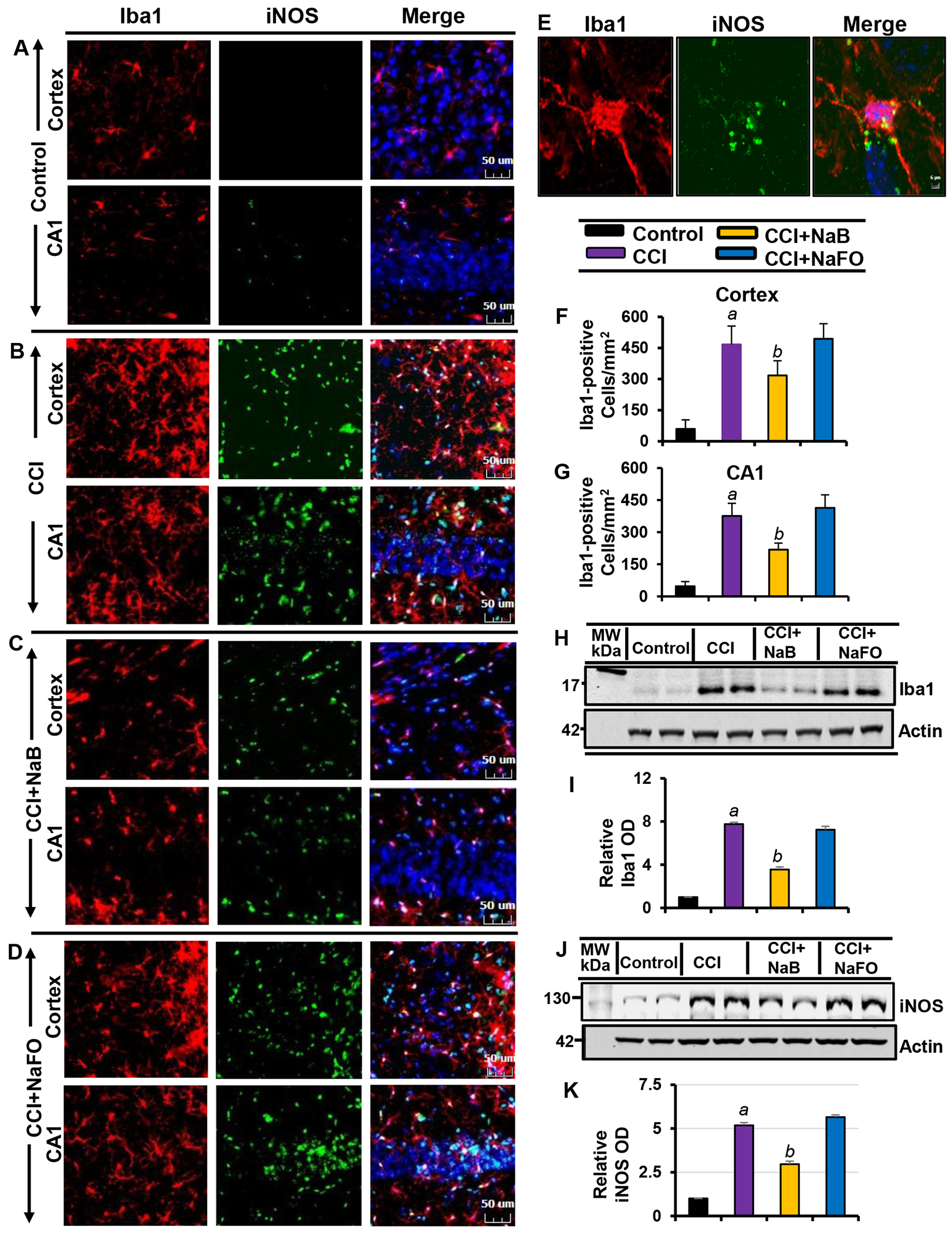
NaB treatment inhibits microglial activation in vivo in the cortex and hippocampus of mice with CCI injury. Mice were treated with 50 mg/kg/day of NaB or NaFO from 24 h after the induction of CCI injury. After 7 d of treatment, brain sections were analyzed by double-label fluorescence for Iba1 and iNOS (A, control; B, CCI; C, CCI+NaB; D, CCI+NaFO). (E) Confocal image demonstrating the co-localization of iNOS and Iba1 in the cortex. Cells positive for Iba1 were counted in the cortex (F) and CA1 region (G). Results represent analysis of six sections of six mice each per group: *^a^ p* < 0.001 vs. control; *^b^ p* < 0.001 vs. CCI. Hippocampal tissue extracts from all groups of mice (n = 4 per group) were immunoblotted for Iba1 (H) and iNOS (J). Actin was run as a loading control. Bands were scanned, and values (I, Iba1/Actin; K, iNOS/Actin) are presented as relative to control. *^a^ p* < 0.001 vs. control; *^b^ p* < 0.001 vs. CCI.

Recent findings have established microglial and astroglial activation and associated neuroinflammation as important pathological events in different neuroinflammatory and neurodegenerative disorders, including brain injury [37,53]. Immediately after the initial CCI injury, the tissue environment is modified to activate glial cells [11,12]. Accordingly, following CCI insult (Figure 1), we observed a marked increase in the number of GFAP-positive astrocytes (Figure 2 A,B,E,F,G) and Iba1-positive microglia (Figure 3A,B,E,F,G) in the cortex and hippocampus region of mice on day 7 post-injury as compared to the sham control. Western blot analysis of hippocampal extracts also corroborated this increase in GFAP (Figure 2J–K) and Iba1 (Figure 3J–K).

The typical oral dose of NaB for adults is 2-5 g twice daily [54,55]. According to Walther et al., no toxicity of NaB treatment was detected in humans when administering doses of up to 470 mg/kg body weight per day [56]. Since NaB reacts with glycine to produce hippuric acid, which is excreted through urine, after oral treatment, a noticeable amount of NaB is used in the body to scavenge glycine. In a number of earlier findings, we found that the neuroprotective effect of NaB is maximal at a dose of 50 mg/kg body wt/d or 100 mg/kg body wt/d [19,21,26,28,52,57]. Therefore, here, CCI-insulted mice were treated with NaB orally via gavage at a dose of 50 mg/kg body wt/d, which led to a decrease in both GFAP-positive astrocytes (Figure 2A–D,F,G) and Iba1- positive microglia (Figure 3A–D,F,G). This result was specific as sodium formate (NaFO) remained unable to inhibit glial activation in the hippocampus and cortex of TBI mice (Figures 2A–D,F,G and 3A–D,F,G).

Decreases in and normalization of the protein levels of GFAP (Figure 2J–K) and Iba1 (Figure 3H– I) in the hippocampus of NaB-treated TBI mice were also evident from Western blots. Activated glial cells are known to express inducible nitric oxide synthase (iNOS) that produces excessive nitric oxide to cause nitrosative stress in a neuroinflammatory milieu [7,58]. Correspondingly, the level of iNOS was higher in the cortex and hippocampus of TBI mice on day 7 post-injury in comparison to the sham control (Figures 2 and 3). Double-label immunofluorescence analysis revealed that increased iNOS was present in both GFAP-expressing astrocytes (Figure 2A–I) and Iba1-positive microglia (Figure 3A–E). However, treatment of TBI mice with NaB, but not NaFO, led to inhibition of iNOS in both the cortex and hippocampus (Figures 2C–D and 3C–D).

These findings were confirmed by quantitative analyses (Figure 2H–I) and Western blotting (Figure 3J–K). Collectively, these results denote that NaB is capable of reducing glial inflammation in vivo in the CNS of CCI-induced TBI mice.

### 3.2. NaB treatment reduced the lesion volume in CCI-induced mice.

**Figure 4:**
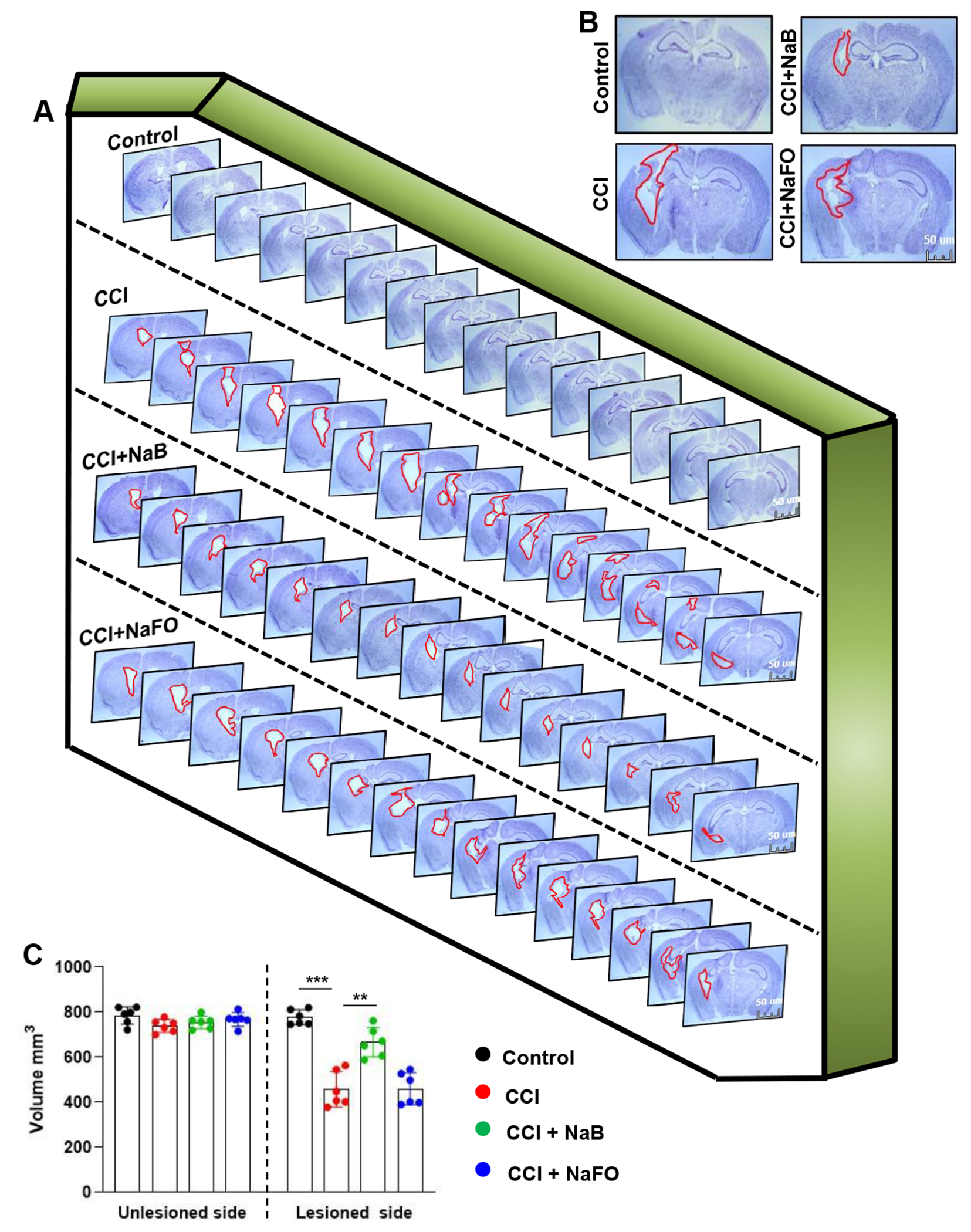
NaB treatment reduces the lesion volume in mice with CCI injury. (A) Representative cresyl violet sections of mouse brain arranged in series of the hippocampal region show the volume of the lesion cavity in different groups. (B) Illustrative images of cresyl violet sections are shown. Note the extent of damage induced in the brain was found to be reduced in NaB-treated mice when compared to CCI mice without treatment and NaFO-treated CCI injury mice. (C) Lesion size was quantitatively measured in control mice, untreated CCI-injured mice, NaB-treated CCI mice and NaFO-treated CCI mice at 21 days post-injury. Statistical analyses were performed with two-way ANOVA and are expressed as mean ± SD to compare the lesion volume 27 between unlesioned and lesioned sides of the brain, as [F1,20 = 156.60 (*** p < 0.0001] control vs. CCI, and [F3,20 = 30.23 (** p < 0.001) CCI vs. CCI+NaB treatment, respectively.

Since oral NaB reduced glial inflammation in the CNS of TBI mice, next, we examined whether NaB treatment was capable of reducing the lesion volume. Therefore, we measured the lesion volume in cresyl violet-stained sections and compared untreated and treated groups. In Figure 4A, cresyl violet-stained brain sections arranged serially to evidence the volume of the lesion cavity from different groups of mice are shown. After 21 days post-injury, we observed typical lesions, including an enlarged cavity, originating from the cortex through the hippocampus and connecting to the lateral ventricle in CCI-induced TBI mice, as compared to none in the sham control (Figure 4B). On the other hand, oral administration of NaB, but not NaFO, reduced the size of the lesion cavity in CCI-induced mice. Quantitative analysis of the lesion volume using the Cavalieri stereological technique revealed that the total lesion volume in the whole hemisphere was significantly reduced after oral treatment of NaB when compared to either untreated or NaFO- treated TBI mice (Figure 4C).

### 3.3. NaB treatment augments motor functions, rotarod performance and gait behavior in CCI- insulted mice

**Figure 5.**
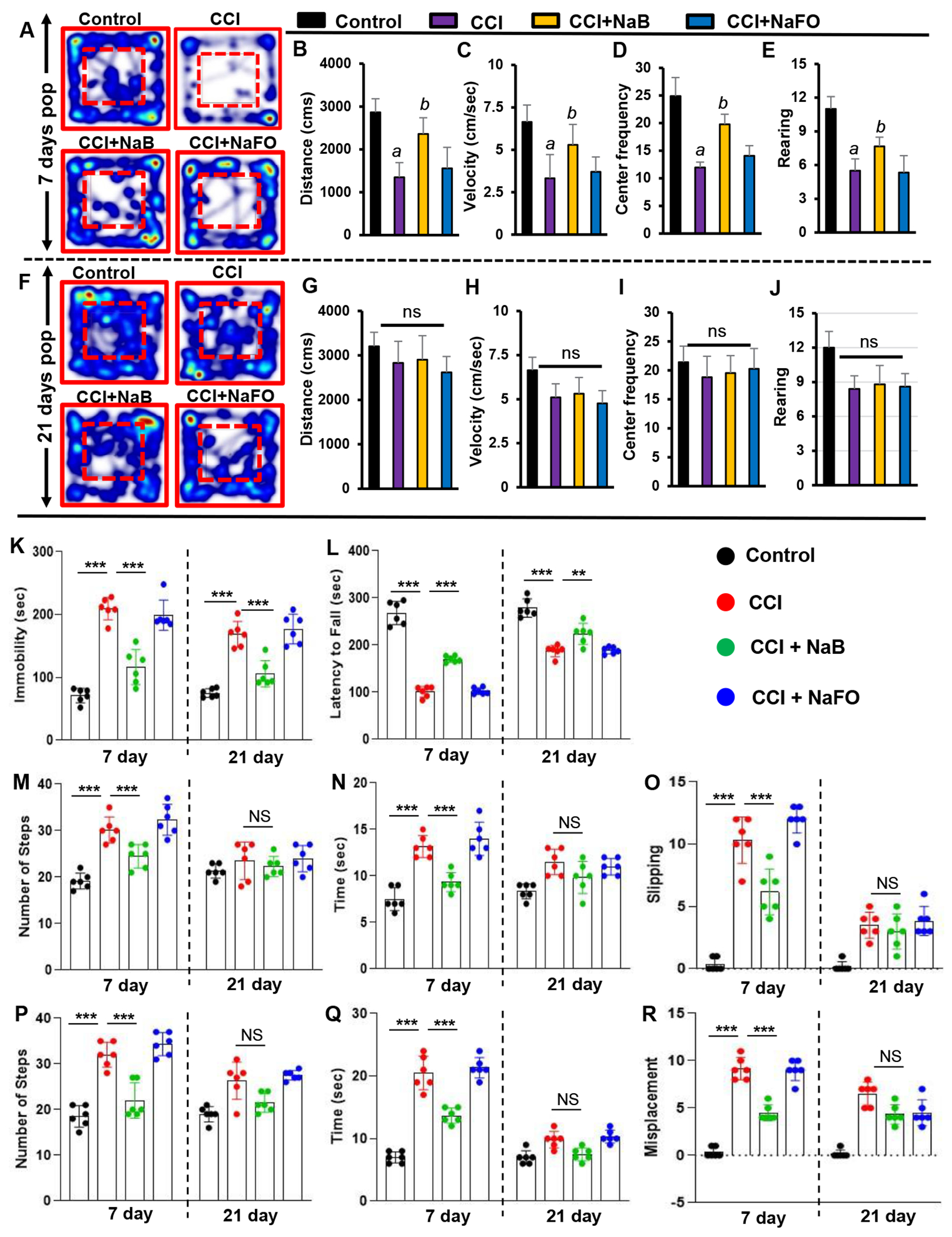
NaB treatment improves motor functions in mice with CCI injury. Mice were orally treated with 50 mg/kg/day of NaB or NaFO from 24 h after the induction of CCI injury. After 7 days of treatment, mice were tested for open field behavior: (A) Heat map analysis was performed by using the Noldus behavioral program; (B) distance moved; (C) velocity; (D) center frequency; and (E) rearing behavior. Statistical analyses were conducted with the Student t-test for distance moved (a p < 0.001 (=0.0001) vs. control; b p < 0.001 (=0.0029) vs. CCI injury); velocity (a p < 0.001 (=0.0001) vs. control; b p < 0.001 (=0.0078) vs. CCI injury); center frequency (a p < 0.001 (=0.0001) vs. control; b p < 0.001 (=0.0036) vs. CCI injury); and rearing behavior (a p < 0.001 (=9.498 × 10–6) vs. control; b p < 0.001 (=0.0081) vs. CCI injury).

The foremost therapeutic objective of neuroprotection research is to limit secondary tissue loss and to preserve or improve behavioral functions. Therefore, to analyze whether oral administration of NaB protected not only the organizational damage but also functional shortages caused by CCI insult, we examined the overall gait activities. A video camera (Basler Gen I Cam, Basler acA 1300-60) connected to a Noldus computer system remained stationary on top facing down on the open field arena for recording general locomotor behaviors. Figure 5A, F represent heat maps summarizing the overall activity of mice in the open field test at 7 days and 21 days post-injury, respectively.

As compared to either untreated or NaFO-treated TBI mice, the general locomotor activity showed a significant improvement in NaB-treated TBI mice at 7 days post-injury (Figure 5A–E). Functional upgrading was clearly visible from the distance traveled (Figure 5B), velocity (Figure 5C), center frequency (Figure 5D) and rearing behavior (Figure 5E). On the other hand, we did not observe significant differences in overall movements between treated and untreated TBI groups at 21 days post-injury (Figure 5).

Subsequently, we also examined the recovery of motor coordination and balance activity in all groups of CCI-insulted mice using the rotarod test at 7 days and 21 days post-injury. Following CCI injury, mice without treatment showed a significant decrease in latency to fall at 7 days post-injury, and this motor activity remained impaired in the rotarod test throughout the 21 days post-injury as compared to the sham control group. However, treatment of CCI-injured mice with NaB, but not NaFO, resulted in prolonged latencies by maintaining the proper body movements and balancing functions in the rotarod test (Figure 5L).

Depression is a common symptom noticed during the initial stage of brain injury. Therefore, next, we monitored depression-like behavior in CCI-injured mice. Previous studies in TBI research have demonstrated that depression in mice can be analyzed by an increase in the duration of immobility [45]. Hence, we performed this test to examine the neuroprotective effect of NaB on depression-like behavior in CCI-insulted mice.

At 7 days post-injury, CCI mice without any treatment showed a significantly longer immobility time than sham controls (Figure 5K). On the other hand, CCI mice treated with NaB exhibited significantly less immobility time compared to either untreated or NaFO-treated mice (Figure 5K). Upon NaB treatment, the duration of immobility was close to the normal level (Figure 5K). These results suggest that NaB is capable of controlling depression-like behavior in CCI- insulted mice.

TBI-induced damage always impairs the connection between the brain and muscles, ultimately affecting gait movements. Consequently, we analyzed gait-related impairments in CCI mice on the beam and grid as these two multifaceted runways appeared to divulge different patterns of movement to those in the open field behavior test. Earlier studies have revealed that these beam and grid runways are particularly useful in models of unilateral TBI because they provide scientists with the opportunity to analyze and compare the contralateral vs. ipsilateral limb movements (Inoue et al., 2013).

Hence, we examined the neuroprotective role of NaB in the recovery of gait functions in our unilateral CCI model using beam and grid runways. CCI mice tended to drag the contralateral pelvic limb while walking. This type of behavior was not seen in sham controls. Further, sham controls did not show significant changes in the latency or number of footsteps to cross the beam after surgery. However, none of the CCI mice were able to cross the beam on the day of surgery and the day after surgery (Figure 5M–O). On day 7 post-injury, CCI mice without treatments showed significant deficits in balancing their bodies on the beam and paw slipping through the grid. CCI mice without treatments showed poor performance in gait behavior, exhibiting more latency, steps and foot faults, or foot misplacement, while crossing the beam (Figure 5M–O), as compared to sham controls. Similar results were seen in the grid analysis (Figure 5P–R). However, upon treatment with NaB, but not NaFO, CCI-injured mice demonstrated significant improvement in gait movement on the beam (Figure 5M–O) and grid runways (Figure 5P–R).

NaB-treated CCI mice also exhibited a significant upgrade in latency, footsteps, foot slips and foot misplacement as compared to either untreated or NaFO-treated CCI mice (Figure 5M–R). On the other hand, at 21 days post-injury, CCI mice recovered considerably to the near-normal level as we did not see significant changes in these parameters with respect to sham controls. As a result, NaB treatment also did not display significant protection either in beam walking (Figure 5M–O) or on grid runways (Figure 5P–R) in CCI mice at 21 days post-injury.

### 3.4. NaB treatment protects spatial learning and memory in CCI-injured mice

Survivors of brain injury usually suffer from learning and memory dysfunctions throughout the rest of their lives [59]. The hippocampus is a major component of the brain that is involved in memory and spatial learning behavior. Therefore, to examine whether NaB protects memory and cognitive functions in our CCI-induced TBI model, we analyzed the mouse behavior in the Barnes maze, T-maze and novel object recognition (NOR) tests.

**Figure 6:**
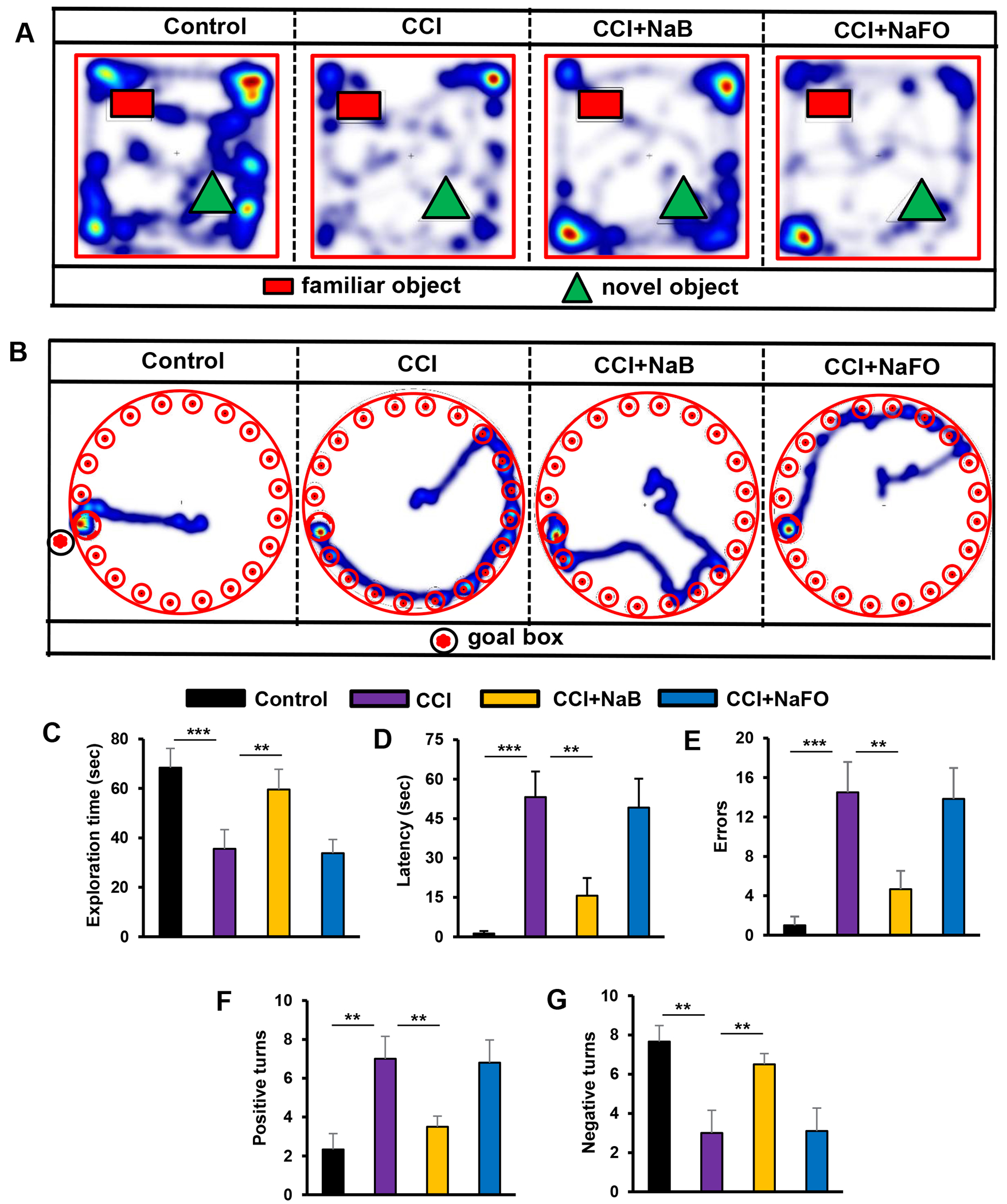
Effect of NaB treatment on spatial learning and memory in mice with CCI-injury. Mice were treated with NaB or NaFO orally (50 mg/kg/day) from 24 h after the induction of CCI injury. Following 21 days of treatment, mice were tested by the novel object recognition test (A, heat map; C, exploration time), Barnes maze (B, heat map; D, time taken; E, number of errors) and T-maze (F, positive turns; G, negative turns). Six mice were used in each group. Statistical analyses were performed by one-way ANOVA followed by Tukey’s post hoc test for the novel object recognition test (exploration time: (*** p < 0.0001) vs. control; (** p < 0.0099) vs. CCI injury); Barnes maze test (time taken: (*** p < 0.0002) vs. control; (** p < 0.0014) vs. CCI injury, and number of errors: (*** p < 0.0006) vs. control; (** p < 0.0012) vs. CCI injury); and T-maze (positive turns: (** p < 0.0063) vs. control; (** p < 0.0035) vs. CCI injury, and negative turns: (** p < 0.0064) vs. control; (** p < 0.0035) vs. CCI injury).

The Barnes maze, a hippocampus-dependent memory task, requires spatial reference memory. Post hoc tests of multiple comparisons analysis showed that CCI-wounded mice without treatments did not find the reward hole easily; they required more time (latency) and made more errors as compared to sham control mice. On the contrary, NaB-treated, but not NaFO-treated, CCI-insulted mice were as capable as sham control mice in finding the target hole, with less latency and fewer errors (Figure 6B,D,E). Similarly, in the T-maze test, CCI-wounded mice without treatments displayed a lower number of positive turns and a higher number of negative turns than sham control mice (Figure 6F,G). On the other hand, oral treatment with NaB, but not NaFO, significantly improved the hippocampus-dependent memory performance in CCI-injured mice as exhibited by a higher number of positive turns and a lower number of negative turns than untreated CCI-insulted mice (Figure 6F,G).

Finally, we examined the short-term memory behavior by the novel object recognition (NOR) task, which was also significantly lower in CCI-injured mice without treatments as compared to the sham control (Figure 6A,C). On the other hand, treatment of CCI-wounded mice with NaB, but not NaFO, led to significant improvement in short-term memory (Figure 6A) as evidenced by the discrimination index (i.e., the difference between time spent exploring novel and familiar objects during the test phase, Figure 6C).

## 4. Discussion

Although TBI is a major cause of death and disability in the US, despite intense investigation, no effective treatment has been available until now to improve the quality of life in patients with TBI, except for regular medical evaluation and care. Therefore, describing a safe and effective therapy to modulate the pathological process of TBI, resulting in improvement in behavioral outcomes, is an important area of research. The cinnamon metabolite sodium benzoate (NaB) is a widely used food preservative due to its antimicrobial properties [20,27]. Moreover, NaB is an FDA- approved drug for urea cycle disorders and glycine encephalopathy [27,60]. Several pieces of evidence outlined in this study clearly support the conclusion that NaB is capable of suppressing the disease process of TBI in a CCI-induced mouse model. While TBI caused a massive lesion cavity, oral NaB treatment started from 24 h after the CCI decreased the lesion volume and restored the structural tissue integrity of the damaged hippocampus. In contrast, treatment with NaFO, a NaB analog without the benzene ring, remained unable to exhibit such protection. NaB treatment also reduced the depression-like behavior, attenuated motor dysfunction and enhanced cognitive performance in mice with TBI. Furthermore, consistent with its safety track record [25,61,62], oral NaB did not cause any side effects (for example, decrease in body weight, loss of hair, fecal boli, infection or untoward behavior). These results suggest that oral NaB may be beneficial for treatment of TBI and that NaB should not be toxic for TBI patients.

Glial activation and upregulation of proinflammatory molecules in the CNS participate in the pathogenesis of a number of neurodegenerative diseases including TBI [11,12,63]. It is known that immediately after TBI, microglia and astroglia in the brain are activated to produce proinflammatory cytokines (e.g. IL-1β, TNFα, etc.), proinflammatory enzymes (e.g. inducible nitric oxide synthase or iNOS), reactive oxygen species, etc., in toxic amounts for a prolonged time period to ultimately cause axonal damage [7,11,15]. Here we have demonstrated that NaB treatment reduces the level of microglial marker Iba1 and astroglial marker GFAP and decreases the expression of iNOS in the hippocampus of mice with TBI. Therefore, although NaB treatment started from 24 h after TBI in a therapeutic mode, it is capable of reducing and/or normalizing glial inflammation in TBI mice.

The signaling mechanisms by which glial cells are activated are poorly understood. It is reported that NaB inhibits LPS-induced expression of iNOS and proinflammatory cytokines in microglia [64]. TLR4 is a prototype receptor for LPS. However, NaB has no effect on the level of TLR4 in LPS-stimulated microglia, indicating that NaB deters LPS-induced expression of proinflammatory molecules without involving its receptor TLR4 [64]. Interestingly, intermediates (HMG-CoA, mevalonate and farnesyl pyrophosphate), but not the end products (cholesterol and coenzyme Q), of the mevalonate pathway reverse the anti-inflammatory effect of NaB in microglia [64].

Suppression of LPS-induced activation of NF-κB and expression of iNOS in glial cells by farnesyltransferase inhibitor proposes an important role of farnesylation reaction in the upregulation of iNOS gene [64,65]. Consistent to a role of farnesylation in the activation of p21ras, it is seen that p21ras signaling plays an important role in the expression of proinflammatory molecules in glial cells [65]. Therefore, suppression of p21ras activation in microglial cells by NaB indicates that NaB attenuates glial inflammation via suppression of p21ras activation.

Until now, no effective interdictive therapy is available for stopping the progression of TBI. Although anticoagulants are there to prevent blood clots and improve blood flow, anti-anxiety medications for reducing fear and nervousness, antidepressants to treat symptoms of depression and mood instability, anticonvulsants for preventing seizures, muscle relaxants to decrease muscle spasms, except anticoagulants others are peripheral treatments. Moreover, some of these medications show limited symptomatic relief exhibiting a number of side effects. On the other hand, there are several advantages of NaB over available TBI therapies. *First*, NaB is objectively safe. It is water soluble and if consumed in excess, it is secreted through the urine. NaB is an FDA-approved drug against urea cycle disorders and nonketotic hyper glycinemia in children [27,60]. Moreover, cinnamon, a commonly used as flavoring material and spice throughout the world for centuries, is also metabolized to NaB. *Second*, it is nice to mention that NaB can be taken orally, the least painful route of drug treatment. Consistent to that observed in animal models of multiple sclerosis, Parkinson’s disease, Alzheimer’s disease and Huntington disease here, oral NaB reduced glial activation *in vivo* in the hippocampus and improved cognitive performance in TBI mice [21,26,28,30,31,52,57,66]. *Third*, NaB is economical compared to the other existing anti-TBI therapies. *Fourth*, entry of drugs through the blood-brain barrier (BBB) is an important issue for the treatment of CNS disorders. Although in the early phase of TBI, the BBB remains compromised, with time, the integrity of BBB improves and therefore, BBB-permeable drugs will be definitely helpful for neuroprotection in TBI patients. In patients with urea cycle disorders, upon oral administration, NaB is seen to react with glycine to produce hippurate, a compound that is readily released in the urine. Simultaneous investigations of serum and CSF samples of these hyperammonemia patients displayed comparable levels of hippurate and NaB in the CSF [67,68]. We have also detected NaB in the brain of mice that were treated with cinnamon orally [19,52]. Therefore, after oral treatment, NaB enters into the brain.

In summary, we have established that oral administration of NaB, a metabolite of cinnamon and a commonly used food additive, decreases glial inflammation, reduces lesion cavity, and improves motor and cognitive functions in a preclinical model of TBI. Although CCI- induced mouse model of TBI may not truly recapitulate the *in vivo* situation of axons in the brain of TBI patients, our results highlight an important neuroprotective effect of NaB and suggest that NaB may be repurposed for therapeutic intervention in TBI.

## Figures and Tables

**Table 1:** Antibodies, sources and dilutions used in this paper

Antibody	Manufacturer	Catalog	Host	Application/Dilution
GFAP	Dako	Z0334	Rabbit	IF/1:2000
iNOS	BD Biosciences	610432	Mouse	IF/1:500
Iba1	Abcam	ab5076	Goat	IF/1:500
GFAP	Dako	Z0334	Rabbit	WB/1:1000
iNOS	BD Biosciences	610432	Mouse	WB/1:1000
Iba1	Abcam	ab5076	Goat	WB/1:1000
Actin	Abcam	ab1801	Mouse	WB/1:5000

IF, immunofluorescence; WB, western blot; GFAP, glial fibrillary acidic protein; iNOS, inducible nitric oxide synthase; Iba1, ionized calcium-binding adapter molecule 1.
